# Myxedema Coma: A New Look into an Old Crisis

**DOI:** 10.4061/2011/493462

**Published:** 2011-09-15

**Authors:** Vivek Mathew, Raiz Ahmad Misgar, Sujoy Ghosh, Pradip Mukhopadhyay, Pradip Roychowdhury, Kaushik Pandit, Satinath Mukhopadhyay, Subhankar Chowdhury

**Affiliations:** Institute of Post-Graduate Medical Education & Research, Calcutta 700020, India

## Abstract

Myxedema crisis is a severe life threatening form of decompensated hypothyroidism which is associated with a high mortality rate. Infections and discontinuation of thyroid supplements are the major precipitating factors while hypothermia may not play a major role in tropical countries. Low intracellular T3 leads to cardiogenic shock, respiratory depression, hypothermia and coma. Patients are identified on the basis of a low index of suspicion with a careful history and examination focused on features of hypothyroidism and precipitating factors. Arrythmias and coagulation disorders are increasingly being identified in myxedema crisis. Thyroid replacement should be initiated as early as possible with careful attention to hypotension, fluid replacement and steroid replacement in an intensive care facility. Studies have shown that replacement of thyroid hormone through ryles tube with a loading dose and maintenance therapy is as efficacious as intravenous therapy. In many countries T3 is not available and oral therapy with T4 can be used effectively without major significant difference in outcomes. Hypotension, bradycardia at presentation, need for mechanical ventilation, hypothermia unresponsive to treatment, sepsis, intake of sedative drugs, lower GCS and high APACHE II scores and Sequential Organ Failure Assessment (SOFA) scores more than 6 are significant predictors of mortality in myxedema crisis. Early intervention in hypothyroid patients developing sepsis and other precipitating factors and ensuring continued intake of thyroid supplements may prevent mortality and morbidity associated with myxedema crisis.

## 1. Introduction

Myxedema coma is a severe and life-threatening form of decompensated hypothyroidism with an underlying precipitating factor. The mortality rates may be as high as 25–60% even with best possible treatment [1–5]. The term myxedema coma is a misnomer, and myxedema crisis may be an apt term as quite a few patients are obtunded, rather than frankly comatose. As the disease is rare and unrecognized, we only have a few isolated case reports and case series, and there is a dearth of randomized controlled trials in the field of myxedema crisis. At present there are over 300 cases reported in literature [6–8]. In this paper we discuss the standard clinical presentation, treatment, predictors of mortality, and controversies that overshadow the current concepts in the management of myxedema crisis.

The need of the hour is to find answers to some burning questions which may change the way we manage myxedema crisis. Some of these questions include the following:

- what are the preventable precipitating factors in myxedema crisis?
- are there any geographical variations involved in the presentation?
- how can we identify myxedema crisis at an early stage?
- are there ways by which we can identify patients at risk of dying?
- what is the status of intravenous and oral replacement therapy with T4 and T3?

At present, we need more well-designed studies to address some of these questions. 

### 1.1. Epidemiology

Case series and case reports from western world tell us that the incidence of myxedema crisis is about 0.22 million per year, but there is a scarcity for such epidemiological data from countries that lie around the equator [9]. Epidemiology of myxedema crisis follows the same pattern as in hypothyroidism and is more common in women and elderly.

### 1.2. Precipitating Events

Literature reveals that most cases present in winter, and hypothermia is a common manifestation. Low ambient temperature may alter thermoregulatory mechanisms and hence will lower the threshold for encephalopathy [1]. However, in our own experience, the presentation may be only slightly more in winter months, and the incidence of severe hypothermia may be lower in tropical countries such as ours as temperatures below 10 degree Celsius are rare, and ambient temperature may influence the degree of hypothermia (unpublished data). In our experience quite a few patients with myxedema crisis have temperatures 2-3°F below normal although we also see patients with severe hypothermia. 

Infections and septicemia are the leading precipitating factors [2, 3]. Typical infections include pneumonia, urinary tract infections, and cellulitis. Cerebrovascular accidents, congestive cardiac failure, road traffic accidents, gastrointestinal bleeding, and various sedative drugs may play a role in precipitating myxedema crisis. Diuretics may mask some of the myxedematous features, and they may also aggravate the hyponatremia associated with myxedema crisis.

Recently, Chu and Seltzer reported a case of myxedema crisis precipitated by consumption of raw bok choy [10]. Bok choy or Chinese white cabbage contains glucosinolates. Some of the breakdown products of glucosinolates, such as thiocyanates, nitriles, and oxazolidines, have been implicated for their inhibitory effects on the thyroid as they may inhibit the uptake of iodine. When eaten raw, brassica vegetables release the enzyme myrosinase, which accelerates the hydrolysis of glucosinolates. Cooking deactivates myrosinase.

A commonly ignored background factor in myxedema crisis is the discontinuation of thyroid supplements in critically ill patients. This is possibly due to the fact that attention may be focused on presenting features and precipitating factors, and associated hypothyroidism is generally ignored (Box 1). 

## 2. Pathogenesis of Myxedema Crisis

Low intracellular T3 secondary to hypothyroidism is the basic underlying pathology in myxedema crisis which leads to hypothermia and suppression of cardiac activity. The body tries to compensate by neurovascular adaptations including chronic peripheral vasoconstriction, mild diastolic hypertension, and diminished blood volume. 

Decreased central nervous system sensitivity to hypoxia and hypercapnia leads to respiratory failure [11, 12]. Other factors contributing to respiratory failure include respiratory muscle dysfunction, obesity, pleural effusions, macroglossia, reduced lung volume, myxedema of the nasopharynx and larynx (reduces the effective airway opening), pneumonia, and aspiration [12–14]. 

**Figure 1:**
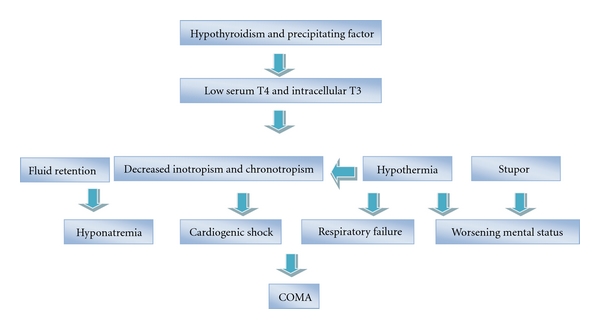
Pathogenesis of myxedema crisis.

Altered vascular permeability leads to effusions and anasarca. Water retention and hyponatremia occurs secondary to reduced glomerular filtration rate, decreased delivery to the distal nephron [15], and excess vasopressin [16, 17] (Figure 1). 

Low intracellular T3 leads to depressed cardiac functions with decreased inotropism and chronotropism with vasoconstriction. The hypothyroid heart tries to perform more work at a given amount of oxygen by better coupling of ATP to contractile events. A precipitating factor pushes this precarious balance over the brink [18]. In the decompensated state, low cardiac output and hypotension will result in cardiogenic shock which may not be responsive to vasopressors without thyroid hormone replacement [11].

Decreased gluconeogenesis, precipitating factors like sepsis and concomitant adrenal insufficiency, may contribute to hypoglycemia. In addition to the generalized depression of cerebral function, hyponatremia, hypoglycemia, hypoxemia, and reduced cerebral blood flow can precipitate focal or generalized seizures and worsen the level of consciousness.

## 3. Clinical Features

A low index of suspicion and a search for precipitating factors should be initial step in dealing with myxedema crisis at an earlier stage. History should focus on the presence of thyroid dysfunction, dose of thyroid hormone, discontinuation of thyroid supplements, thyroid surgery, radioactive iodine ablation, and a detailed record of drug intake for background diseases. Central hypothyroidism may constitute about 5% of all the cases of myxedema crisis [1]. Physical examination should focus on features of severe hypothyroidism like dry skin, sparse hair, a hoarse voice, hypothermia, delayed tendon reflexes, macroglossia, nonpitting edema, goiter, and surgical scar of thyroidectomy in addition to recording vital functions and level of consciousness. Presence of orbitopathy may be a subtle clue to underlying Graves's disease which may have been treated with radioiodine or surgery.

Dutta et al. studied 23 patients with myxedema crisis and found that 39% of them had hypothyroidism detected only at the time of crisis. It should be noted that 17% of these patients had central hypothyroidism which was higher than the previous reported percentage of 5%. Sepsis was the most common precipitating factor, and a significant proportion of patients (61%) had defaulted on thyroid supplements [4]. Reinhardt and Mann reported hypoxemia in 80%, hypercapnia in 54%, and hypothermia with a temperature less than 94°F in 88% of all the patients with myxedema crisis [5]. 

Sinus bradycardia, low voltage complexes, bundle branch blocks, complete heart blocks, and nonspecific ST-T changes in electrocardiogram have been recorded in myxedema crisis [4, 19]. Schenck et al. reported a patient with severe hypothyroidism who presented with presyncope, prolongation of the QT interval, and polymorphic ventricular tachycardia (torsades de pointes) which reversed with thyroid hormone supplementation [20]. Prolongation of QT interval and increased QT dispersion, a marker of electrical instability [21, 22], have been documented in severe hypothyroidism [23]. These anomalies have been shown to be reversible in subclinical hypothyroidism [24]. Increased myocardial fibrosis in severe hypothyroidism may lead to a resistance in improvement of QT dispersion with thyroid hormone supplementation [24]. The study of heart rate variability parameters also indicate that hypothyroidism leads to a sympathovagal imbalanced state, characterized by both decreased cardiovascular sympathetic and vagal modulation [23]. The occurrence of malignant arrhythmias needs to be recognized in long-standing hypothyroidism and myxedema crisis [19, 23, 25] (Table 1). 

An important practical aspect may be the identification of pericardial effusion and myocardial infarction in a setting of myxedema crisis. Low-voltage complexes and nonspecific ST-T changes can be seen in pericardial effusion. Cardiac enzymes should be done with a suspicion of myocardial infarction.

Patients with myxedema crisis may have normocytic normochromic anemia which may be secondary to decreased oxygen requirement and erythropoietin [26]. Macrocytic blood picture may be seen with associated low folate absorption and pernicious anemia [27–29]. Severely hypothyroid patients may have bleeding manifestations, and investigations reveal prolonged bleeding time and clotting time, decreased platelet adhesiveness, elevated APTT, and low or normal factor VIII activity. Acquired von Willebrand's disease is also reported in hypothyroidism with decreased von Willebrand factor antigen and activity [30]. Acquired von Willebrand's disease is very likely to be type 1 in all cases because of a normal ratio of von Willebrand's factor antigen to ristocetin cofactor *r* [31]. The underlying defect could possibly be a decreased synthesis of von Willebrand factor in the absence of adequate levels of thyroxine [32], and thyroxine replacement corrects these abnormalities [31, 33]. Erfurth and group have demonstrated that desmopressin immediately reduced bleeding time, enhanced platelet adhesiveness, and significantly increased plasma concentrations of factor VIII and von Willebrand's factor and hence may be valuable for the acute treatment of bleeding or as cover for surgery in a setting of myxedema crisis or severe hypothyroidism [33]. 

Other common biochemical anomalies in myxedema crisis include increased levels of creatine phosphokinase, lactate dehydrogenase, aspartate transaminase, and hypercholesterolemia (elevated LDL) [34].

## 4. Diagnosis

Patients with altered sensorium, hypothermia, or absence of fever despite infectious disease, clinical and biochemical features of hypothyroidism, those who had a history of hypothyroidism and are currently not on treatment, in the setting of a precipitating factor, should be identified with a high index of suspicion. The treating physician should not hesitate in starting replacement therapy while waiting for serum TSH and serum T4. An active search for precipitating causes should be initiated, and appropriate investigations should be ordered based on patient's clinical presentation. White blood cell counts, urine routine and microscopy, blood and urine culture, serum electrolytes, serum creatinine, chest X-ray, and electrocardiogram should be obtained.

Clinician may face multiple diagnostic dilemmas. First, in central hypothyroidism, TSH may be unusually low. The deciding factor here will be associated pituitary hormone deficiencies as isolated central hypothyroidism is rare. In the second situation, the systemic illness will produce a picture akin to sick euthyroid syndrome, and the elevation in TSH may not be as high as expected, but the T3 levels here may be unusually low as there is decreased conversion of T4 to T3 [34, 35] with elevated reverse T3. Finally, the presenting features of myxedema crisis may also be commonly seen with associated sepsis, stroke, and dyselectrolytemia, and hence the diagnosis is often delayed.

It may be appropriate at this juncture to discuss the rare entity of Hashimoto's encephalopathy which is a rare complication of Hashimoto's thyroiditis [36]. The condition may present as a subacute or acute encephalopathy with seizures, stroke-like episodes, myoclonus, and tremor [37]. Patients will have elevated thyroid-specific autoantibodies (Anti-TPO), elevated cerebrospinal fluid protein without pleocytosis, and abnormal electroencephalogram [38]. The key points to consider here is that most patients are euthyroid and the condition is steroid responsive [39].

## 5. Treatment of Myxedema Crisis

Treatment of myxedema crisis should be prompt and multidimensional with attention to the following principles:

- intensive care treatment with ventilator support, central venous pressure monitoring, and pulmonary capillary wedge pressure if feasible in patients with cardiac disease,
- appropriate fluid management and correction of hypotension and dyselectrolytemia,
- aggressive management of precipitating factors and steroid supplementation if required,
- thyroid hormone replacement.

## 6. General Measures in Management

Management of airway and airway protection from aspiration in case of patients with poor consciousness level should be the utmost priority. Endotracheal intubation or tracheostomy with mechanical ventilation may be performed. Arterial blood gas should be monitored frequently to ensure adequate oxygenation and correction of hypercarbia. Sedatives and other drugs may exacerbate the respiratory depression and may delay the weaning of ventilator support [40].

Fluid management evokes a Damocles sword type situation in myxedema crisis where the choice is between fluid supplementation for hypotension and fluid restriction for hyponatremia. A pragmatic approach in mild hyponatremia will be to advice fluid restriction with replacement to cover the daily losses taking care to supplement glucose, sodium, and potassium [41]. In a situation of severe hyponatremia, it may be prudent to administer 3% sodium chloride along with furosemide, so that serum sodium may be elevated by 3-4 meq/L to tide over the immediate crisis [42]. A rapid correction of chronic hyponatremia might put patients at risk for central pontine myelinolysis [43, 44]. Treatment with furosemide will prevent fluid overloading associated with hypertonic saline. In experimental hypothyroidism, the impaired response to an acute water load was shown to be reversed by a Vasopressin receptor antagonist (V2R antagonist) [17]. Future research with V2R antagonists in treating hyponatremia associated with myxedema crisis may prove to be interesting.

Hypothermia may be managed by external warming, but the accompanying vasodilatation may precipitate hypotension. Hypotension requires careful infusion of dextrose saline solutions and vasopressors if required. A search for other causes of hypotension like sepsis, myocardial infarction, pericardial effusion, and occult bleeding should be initiated. In a setting of concomitant adrenal insufficiency, hydrocortisone supplementation is also required for correction of hypotension.

Hypocortisolemia may be due to primary or secondary adrenal insufficiency. The clinical features of myxedema crisis and cortisol deficiency may overlap. Hyperpigmentation, hyperkalemia, hypercalcemia, and previous history of on and off steroid use must be sought. Thyroid hormone replacement may increase cortisol clearance and may aggravate cortisol deficiency. If the facilities for HPA axis evaluation are not available on an emergency basis, steroid therapy may start and a formal evaluation of axis is done at a later date when patient is stable. Intravenous hydrocortisone is preferred at a rate of 50 mg every 6 hours.

It cannot be overemphasized that the precipitating factors require urgent attention with antibiotics in case of infection, hemodialysis for associated renal failure, and comprehensive care of multiorgan dysfunction.

## 7. Thyroid Hormone Therapy 

Thyroid hormone therapy is the backbone of treatment of patients with myxedema crisis. The main considerations with thyroid hormone replacement are the absorption and distribution of administered hormone preparation, the onset of action and efficacy of the preparation, and finally the safety. At present, oral and intravenous T4 and T3 are used. The major considerations are the dose, route of administration, and frequency of administration.

T4 therapy provides a steady, smooth, and slow onset of action with relatively few adverse events. In many countries, T3 may not be available, but T4 is easily available at hand. T4 therapy avoids major peaks and troughs in body, and values of serum T4 may be easy to interpret [1]. However, T3 is the active hormone in the body, and in a setting of severe illness there may be a decreased conversion of T4 to T3 [34]. Parenteral T4 may be used at a dose of 300–500 *μ*g as bolus to saturate the body pool. The usual protocol then is to continue T4 at a dose of 50–100 *μ*g daily. There are enough studies that have used a large bolus dose of T4 as described earlier, and they demonstrate good results [45, 46]. T4 concentrations rise acutely to levels above normal and slowly gets converted to T3 [46]. 

Oral administration of T4 through Ryles tube has proved to be equally effective with a drawback that gastric atony may prevent absorption and put the patient at risk for aspiration. Dutta and colleagues compared 500 *μ*g of oral loading dose of T4 with 150 *μ*g of maintenance dose orally and 200 *μ*g of T4 intravenously followed by 100 *μ*g T4 intravenously until they regained their vital functions and were able to take oral medications in patients with myxedema crisis and did not find any difference in outcome among the patients [4].

Advantages of using T3 include a rapid onset of action, an earlier beneficial effect on neuropsychiatric symptoms, and significant clinical improvement within 24 hours. T3 may be given at a dose of 10 to 20 *μ*g, followed by 10 *μ*g every 4 hours for the first 24 hours and then 10 *μ*g every 6 hours for 1 or 2 days till the patient is alert enough to continue therapy through oral route. Measurable increases in body temperature and oxygen consumption occur within 2 to 3 hours after intravenous administration of T3 but may take 8 to 14 hours or longer after intravenous administration of T4 [1, 47]. However, poor availability of T3, fluctuations in serum levels of T3, and adverse cardiac effects may limit the use of T3. Yamamoto et al. reported that doses of LT4 more than 500 *μ*g per day and LT3 more than 75 *μ*g/day were associated with increased mortality [3]. 

Combined therapy of T4 and T3 may also prove to be useful. T4 may be initiated at a dose of 4 *μ*g/kg of lean body weight, followed by 100 *μ*g 24 hours later and then 50 *μ*g daily intravenously or orally. T3 may also be started simultaneously with T4 at a dose of 10 *μ*g iv, and the same dose is given every 8 to 12 hours until the patient can take maintenance oral doses of T4 [1].

## 8. Factors Predicting the Mortality in Myxedema Crisis

Dutta and colleagues looked at the predictors of outcome in myxedema crisis and found that hypotension, bradycardia at presentation, need for mechanical ventilation, hypothermia unresponsive to treatment, sepsis, intake of sedative drugs, lower GCS, and high APACHE II scores were significant predictors of mortality. Sequential organ failure assessment (SOFA) score was more effective than other predictive models. Baseline and day 3 SOFA scores of more than 6 were highly predictive of poor outcome. They also demonstrated that treatment defaulters presented early to the hospital and had more severe manifestations than *de novo* patients [4]. 

Rodríguez and colleagues showed that those patients with coma at the time of presentation, low Glasgow coma scale scores, and higher APACHE II scores had considerably poor outcome [2]. Studies have also shown that higher doses of T3 are associated with increased mortality, and lower doses of T3 and T4 may be associated with favorable prognosis [3, 47, 48]. Other factors associated with mortality include advanced age and cardiovascular disease [1, 3].

## 9. Summary

Myxedema crisis is a life-threatening extreme form of hypothyroidism with a high mortality rate if left untreated. Myxedema crisis is commonly seen in older patients, especially women, and is associated with signs of hypothyroidism, hypothermia, hyponatremia, hypercarbia, and hypoxemia. There may be a significant delay in diagnosis which may adversely affect the prognosis. Patients should be provided with intensive care facilities with prompt attention to ventilation, hypotension, hypothermia, steroid replacement, and thyroid hormone supplementation. SOFA scoring system may help us to identify patients at risk of mortality at an earlier stage. Early medical attention in hypothyroid patients developing serious illness especially sepsis and ensuring continuation of thyroid supplements may prevent significant morbidity and mortality.

## Figures and Tables

**Box 1:**
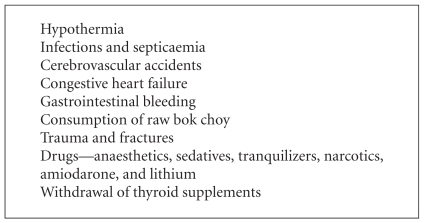
Precipitating factors of myxedema crisis.

**Table 1:** Clinical and laboratory features of myxedema crisis.

Cardiovascular	Neuropsychiatric
Bradycardia and hypotension	Confusion and obtundation
Cardiomegaly	Lethargy
Low cardiac output	Coma
Pericardial effusion	Seizures
Cardiogenic shock	Poor cognitive function
Bundle branch blocks and arrhythmias	Depression and psychosis
Nonspecific ECG findings	

Respiratory	Renal and water metabolism

Hypoxia	Fluid retention
Hypercarbia	Anasarca
Myxedema of larynx	Hyponatremia
Pleural effusion	Bladder atony
Pneumonia (precipitating factor)	Urine sodium normal or increased
	Urine osmolality > serum osmolality

Gastrointestinal	Metabolic

Anorexia and nausea	Hypothermia
Abdominal pain	Hypoglycemia
Constipation	
Paralytic ileus	
Toxic megacolon	
Gastric atony	
Neurogenic oropharyngeal dysphagia	
